# Evaluation of Factors Affecting Clinical Outcomes of Full Mouth Rehabilitation Under General Anaesthesia for Children With Early Childhood Caries: A Prospective Cohort Study

**DOI:** 10.7759/cureus.46778

**Published:** 2023-10-10

**Authors:** Mebin George Mathew, Ganesh Jeevanandan

**Affiliations:** 1 Pediatric and Preventive Dentistry, Saveetha Dental College and Hospitals, Chennai, IND; 2 Pediatric and Preventive Dentisty, Saveetha Dental College, Saveetha University, Chennai, IND

**Keywords:** relapse, oral health related quality of life, comprehensive dental treatment, full-mouth rehabilitation, quality of life, general anesthesia, early childhood caries, clinical outcome, caries recurrence

## Abstract

Aim: This study was designed to evaluate the factors affecting the clinical outcomes of full mouth rehabilitation under general anaesthesia for children with early childhood caries (ECCs).

Materials and methods: A prospective cohort of 200 children with early childhood caries and requiring dental rehabilitation under general anaesthesia was evaluated and treated. Children were recalled at six-month intervals for a period of two years and evaluated for the recurrence of caries and the need for repeat treatment of failed cases.

Results: 86.5% of the patients adhered to the six-month recall visits for 24 months. An overall caries recurrence rate of 14.5% was seen. Children who did not adhere to the follow-up plan and visited ad hoc had the highest caries recurrence rate (88%).

Conclusion: Good compliance with postoperative instructions after full mouth rehabilitation resulted in good oral hygiene and a limited recurrence of caries. Patients with poor compliance with recall visits and postoperative instructions had high rates of caries recurrence. Rehabilitation under general anaesthesia can be considered a viable treatment option for children diagnosed with early childhood caries.

## Introduction

Early childhood caries (ECC) is a form of dental decay that affects primary dentition. Despite being preventable, ECC is now one of the most prevalent diseases affecting infants and preschoolers across the globe and is now considered a major health problem. ECC affects the child's general and oral health and has been shown to affect growth and development [1,2].

ECC has a multifactorial aetiology and is believed to occur because of microbial interaction with sugary food on the primary tooth surface. Various biological, environmental, and psychosocial factors have also been found to influence the development and progression of ECC [3,4]. ECC initiates as a white spot lesion on the labial surfaces of the deciduous incisors. At this stage, ECC is reversible if preventive measures are undertaken and oral hygiene is improved. Children do not complain of pain, and parents do not usually report to the dental office at this stage [5]. Parents often report to the dental office when children have pain or abscesses, by which time ECC has rapidly spread to involve both maxillary and mandibular primary molars. Children may also complain of difficulty eating and smiling at this stage [6].

Because children with ECC are very young and often uncooperative, they may not always be ideal candidates for treatment on the dental chair. To provide the best treatment, children with ECC are often treated under general anaesthesia. It is carried out as a day-care procedure, and full mouth rehabilitation can be completed in a single appointment. Both parents and children have reported high satisfaction with full-mouth rehabilitation under general anaesthesia [7,8].

The oral health-related quality of life has been reported to improve considerably after the completion of dental rehabilitation under general anaesthesia. Despite the high success rates, long-term results have shown that caries recurs in many children. A caries recurrence rate of 79% was reported by Almeida et al. [9], and 17% of these children required treatment to be performed again under general anaesthesia. A six-year follow-up study by Kakaounaki et al. [10] found 8.9% of children required retreatment under general anaesthesia. Though the rate of caries recurrence has been investigated, the factors affecting the clinical success rates of treatments done under general anaesthesia have scarcely been reported. Hence, this study was undertaken to explore factors affecting the clinical success of treatment for early childhood caries under general anaesthesia.

## Materials and methods

This prospective cohort study was initiated after receiving approval from the institutional ethical committee of Saveetha Dental College and Hospitals (IHEC/SDC/FACULTY/21/PEDO/239).

Children younger than 71 months of age who were diagnosed with ECC and who required comprehensive dental treatment under general anaesthesia were included. Children who were outside the age group, who cooperated with dental treatment in the dental operatory, and who had compromised medical issues were not included in this study. Any child who required a repeat treatment under general anaesthesia was not a part of the study.

From a total of 487 patients who visited the Department of Paediatric and Preventive Dentistry for ECC, 200 who fulfilled the criteria and whose parents agreed to partake in the investigation were included.

The selected children were examined in the dental office. Radiographs were taken based on the clinical needs of each patient, and the treatment plan was made based on correlations with the clinical scenario. Parents were informed about the need for dental rehabilitation and told that the treatment would take place in the operating room. Children were sent for blood tests and an echocardiogram, after which a pre-anaesthetic check-up was done. Once the child was deemed fit for the procedure, the date for surgery was fixed. All participants were treated in the paediatric dentistry operation theatre in the dental hospital. The standardised treatment protocol followed by Mathew et al. was used in the present study [11]. Demographic details were collected from parents on the day of surgery.

Participants and their parents were taught about maintaining oral hygiene and were given dietary counselling. All instructions were provided in pamphlets to which the parents could refer at any time. A diet diary was given to each child so that their diet could be monitored. Toothbrushing was demonstrated on a model, after which the child had to demonstrate it back to the operating team to ensure that both parents and the child understood the technique. Parents were also taught brushing and asked to keep track of their child’s brushing. Children were recalled for an early follow-up visit 10 days after the treatment. Children were recalled once at a six-month interval for check-ups and followed up until 24 months had passed.

During the early postoperative visit, occlusion was rechecked, and each child was assessed for the need for further medications. The toothbrushing technique was demonstrated and assessed for both children and parents, followed by diet counselling.

At the six-month follow-up, the detection and immediate treatment of new carious lesions were done using Decayed, Missing, and Filled Teeth (DMFT) criteria. All restorations were monitored to see whether they were successful or failed. Fluoride varnish (Voco Profluorid Varnish, VOCO Dental, Cuxhaven, Germany) was applied at each visit. Oral hygiene and nutritional habits were reinforced on the same visit. Clinical photographs were taken at early postoperative and biannual visits and uploaded into the digital archival information system during each visit.

All patient records were statistically analyzed to assess the relationship between compliance with the postoperative recall plan and caries recurrence as a determinant of the postoperative outcome. Any child who required repeated dental treatment under general anaesthesia was considered a failure.

Based on the postoperative recall plan, patients were divided into the following groups. Group 1: patients who came in for all follow-up visits; group 2: patients who attended the early postoperative visit and one follow-up during the 24-month study period; group 3: patients who did not come in for the early postoperative visit but presented to the dental office for one follow-up visit during the 24-month study period; group 4: patients who did come in for the scheduled follow-up but who also paid impromptu visits during the twenty-four-month study period; group 5: those who did not visit after treatment and were not considered in the study.

## Results

The present investigation consisted of 200 children who required comprehensive dental treatment under general anaesthesia. Participants included 103 boys (51.5%) and 97 girls (48.5%). Of these children, 91.5% belonged to ASA I, and the remaining 8.5% were ASA II; 54% of the children had preoperative DMFT scores between 7 and 13. Of their parents, 68.5% had secondary education or below, whereas 31.5% had postsecondary education or higher, and 56% had an annual family income of less than 2 lakhs a year. The demographic data of the participants and their parents is summarised in Table 1.

**Table 1:** Parent and child characteristics

Variables	N (%)
Gender	
Male	103(51.5)
Female	97(48.5)
Health status (ASA classification)	
ASA I	183(91.5)
ASA II	17(8.5)
Education level of parents	
Secondary or below	137(68.5)
Post-secondary or above	63(31.5)
Family income (per year)	
<200,000	112(56)
200,000–500,000	37(18.5)
>500,000	51(25.5)
Child’s dental status	
DMFT	
<7	27(13.5)
7–13	108(54)
>14	65(32.5)

Table 2 shows the caries recurrence with compliance to postoperative preventive plan attendance: a 16.4% caries recurrence was seen in group I, 41.7% in group II, 66.7% in group III, and 88.9% in group IV. An overall caries recurrence rate of 14.5% was seen in the study population.

**Table 2:** Caries recurrence versus compliance with post-operative preventive plan attendance

	Patient number	Dental caries recurrence	%
Group I	173	12	16.4
Group II	12	5	41.7
Group III	6	4	66.7
Group IV	9	8	88.9
Total	200	29	14.5

The need for repeat treatment is shown in Table 3. One child each in groups I and II required repeat treatment. Three children in group III and six children in group IV also required repeated treatment.

**Table 3:** Need for repeated dental rehabilitation under general anesthesia

	Group I	Group II	Group III	Group IV
Required repeat general anesthesia	1	1	3	6
Did not require repeat general anesthesia	172	11	3	3

Table 4 shows the dental needs and treatment details of the participants in the study. Pulp therapy (55%) was the most common treatment for baseline treatment compared to restorations for repeat treatment. A highly statistically significant difference (<0.001) was seen between all treatment needs at baseline and repeat visits.

**Table 4:** Dental needs and treatment details

Treatment	Baseline details n(%)	Repeat treatment details n(%)	P-value
				Extractions	708(26.4)	24(21.4)	<0.001
Restorations	498(18.6)	47(42)	<0.001
Pulp therapy	1473(55)	32(28.6)	<0.001
Total	2679	112	<0.001

## Discussion

ECC is a significant public health issue that is affecting preschoolers around the world. More than 600 million children have been reported to be suffering from ECC globally [12].

Dental plaque is a major cause of the initiation of ECC. The early colonization by cariogenic bacteria plays an important role in the progress of the disease. ECC spreads rapidly, initially from the primary maxillary incisors and spreading to the maxillary and mandibular primary molars at a later stage [12]. Patients visit their dentist when they have pain and difficulty eating. Owing to their age and the severity of ECC, children usually require aggressive treatment, which is necessary to improve their oral health-related quality of life [13]. Chair-side dental treatment can be attempted, but because of the extent and severity of ECC, treatment may require multiple sittings, and the cooperative ability of the child may vary, which can prolong and affect the clinical success of the treatment [14]. To provide optimal treatment to ensure proper growth and development and improve the oral health-related quality of life, children are treated comprehensively under general anaesthesia. All surgical, endodontic, and preventive treatments are done in a single sitting, and patients are discharged on the same day. Considerable improvement in the oral health-related quality of life after full mouth rehabilitation has been accepted with high satisfaction rates by parents [7,8].

Despite the high success rates of complete dental rehabilitation, long-term results have shown the recurrence of caries. Although it is completely preventable, the prevalence of ECC is rising and has been found to be 48%, with variations seen between and within countries [15]. Even when various preventive measures are taken after full mouth rehabilitation under general anaesthesia, relapse of caries has been reported by various authors, with some patients even reporting the need for retreatment under general anaesthesia [9,10,16]. Hence, full-mouth dental rehabilitation under general anaesthesia is not the main treatment protocol for ECC; rather, an amalgamation of preventive changes in eating habits, oral health practices, and regular dental check-ups after treatment is preferred [2,3,14].

Researchers have expressed differences of opinion about full-mouth rehabilitation for ECC. Graves et al. [17] opined that treatment under general anaesthesia did not produce acceptable treatment results. Amin et al. [18] concluded in 2006 that full-mouth rehabilitation under general anaesthesia resulted in improved dental health practices. Almeida et al. [9] concluded that aggressive preventive therapies are necessary because children with ECC are highly predisposed to developing caries in the future. The present study is in accordance with these opinions because successful clinical outcomes were obtained, but only when stringent compliance with postoperative instructions and preventive interventions were followed.

In the current study, 86.5% of patients returned for follow-up for the complete study protocol. This is a high recall rate compared to previously published studies [9,16-21].

A Saudi Arabian study published by Al-Hussyeen [19] reported that 18.7% returned for at least one postoperative visit other than the immediate postoperative visit. Foster et al. [20] reported a 90% recall rate at one biannual follow-up session, but only 39.4% of patients attended the immediate postoperative visit, whereas 63% of the patients made unscheduled visits during the 24-month study for various treatment needs. El Batawi [16] reported that 14% of the study population attended all follow-up visits, whereas 18% of the patients did not report back for treatment. Of this population, 26% reported during the study, but these were ad hoc visits for treatment needs. A plausible reason for parents to miss follow-up visits could be that the comprehensive treatment along with preventive measures was completed in a single visit, and a significant improvement in oral function and a significant reduction in pain resulted in an improved oral health-related quality of life that would have relieved the common complaints of pain and difficulty eating [2,13,14]. Most children report that when pain occurs, their parents and caregivers ignore preventive treatments such as routine dental care [6]. In the present study, we were able to obtain a high recall rate because the families of the participants were reminded through phone calls and messages to attend the follow-up visits in person.

The overall recurrence rate of caries in the present study was 14.5%. The lowest recurrence was seen in children who visited for all follow-up visits (16.4%), whereas children who visited ad hoc or outside the follow-up visits had the maximum recurrence (88.9%). The recurrence rate for caries reported in the present study was much lower than those reported by Graves et al. (37%) [17], Berkowitz et al. (39%) [21], Foster et al. (58.8%) [20], and Almeida et al. (79%) [9]. The difference in recurrence rates could be attributed to the multifactorial aetiology and complexity with which ECC occurs [16]. The inherent harbouring of various caries-promoting microorganisms, such as Streptococcus mutans and Candida albicans, can promote a cariogenic oral biofilm on an unaffected tooth surface that could result in new carious lesions [22]. Preformed dental crowns have also been found to accumulate plaque, with stainless steel crowns accumulating more plaque than zirconia crowns, which can again be a source of the nidus of infection [23].

The highest rate of recurrence was seen in patients who did not adhere to the preventive follow-up visits. This is in accordance with Sheller et al. [24] and El Batawi [16], who found that patient compliance with postoperative instructions and oral hygiene was the key factor in preventing the occurrence of new carious lesions. Various factors contributing to the occurrence of carious lesions after comprehensive dental treatment done under general anaesthesia have been identified by different authors. Sheller et al. found that children who had been under general anaesthesia for dental treatment before the eruption of primary second molars would eventually require retreatment under general anaesthesia [25]. Sheller et al. [24] found that children treated under general anaesthesia at a young age were always at high risk for retreatment in a short span of time. A study by Landes and Bradnock concluded that children who had extractions under general anaesthesia before four years of age were likely to be candidates for retreatment [26]. The overall repeat rate for treatment under general anaesthesia in this study was 5.5%, which was more than that reported by El Batawi for the same follow-up period of 24 months. However, in the present study, none of the patients were lost to follow-up, compared to the 18.3% loss of patients in the study conducted by El Batawi [16]. Kakaounaki et al. found an 8.9% retreatment rate in a six-year follow-up study [10]. Albadri reported an 11.9% recurrence rate over a three-year period [27]. A possible reason for the low retreatment rates compared with other studies could be the continuous recall visits and biannual fluoride application. A common benefit of full-mouth rehabilitation is that all treatments are completed in a single visit, and the patient does not usually require postoperative dental treatment; if this is required, it can be completed on the dental chair [2,13].

A major change in treatment needs was seen from baseline to the need for repeat general anaesthesia treatment. Pulp therapy decreased from 55% at baseline to 28.6% at visits for repeat general anaesthesia. Extractions decreased from 26.4% to 21.4%, but restorations increased from 18.6% to 42% when baseline and repeat general anaesthesia treatment needs were compared. Our results resembled those of Sheller et al. [24] and El Batawi [16], who found that pulp therapies were highly successful.

The aetiology of dental caries is complex, and that of ECC is even more complex because parents, grandparents, and caregivers play roles in the diet and oral hygiene practices of the child [1,4-6]. Despite the best efforts of the dentist and the dental team in taking preventive measures, the main responsibility for continuing the new changes is in the hands of parents or caregivers. In the present study, a biannual application of fluoride varnish was done to prevent the formation of new carious lesions. Despite repeated demonstrations of correct brushing technique coupled with reinforcement of good oral hygiene practices for both parents and children, new carious lesions were found. This result is similar to the findings of a study by Jiang et al. [28] that described supervised brushing and biannual fluoride application in children in Hong Kong in areas where water was fluoridated. A recent systematic review published by Soares et al. did not find adequate evidence to support the effectiveness of various strategies for the prevention of ECC [29].

Our study had a few limitations. The DMFT scores would bear little or no relation to the number of carious teeth because each tooth would have received treatment and would have changed from decayed to missing or filled. Because complete treatment is finished in a single appointment, if any postoperative treatment is necessary and the patient visits elsewhere, it could bring a change in dental status. The socioeconomic factors affecting ECC in the present study may be related to India and may not be comparable to other countries. The strengths included a good retention rate for patients who reported for all scheduled visits and for long-term follow-up. Though some patients did not complete all the scheduled visits, all patients made a visit during the study period.

## Conclusions

Within the limitations of our study, it was found that good compliance with postoperative instructions after full mouth rehabilitation resulted in good oral hygiene and a limited recurrence of caries. Patients with poor compliance with recall visits and postoperative instructions had high rates of caries recurrence. This emphasises the crucial role of patient compliance in averting caries recurrence. Patients who diligently adhered to postoperative instructions and attended scheduled recall visits demonstrated enhanced oral hygiene and a significantly reduced caries recurrence rate.

These insights underscore the multifaceted nature of ECC and the necessity for a comprehensive, multidimensional approach to its management. While full-mouth rehabilitation under general anaesthesia promptly alleviates symptoms and augments the oral health-related quality of life, long-term success hinges on sustained patient education and involvement in preventive measures. Hence, continued endeavours to educate both caregivers and children about optimal oral hygiene practices and dietary habits are imperative. By seamlessly integrating these aspects into clinical practice, we can optimise the enduring outcomes of ECC treatment, ultimately promoting superior oral health and overall well-being for the afflicted children.
